# Diabetes mellitus is an independent predictor of right ventricular dysfunction

**DOI:** 10.1186/1532-429X-17-S1-P147

**Published:** 2015-02-03

**Authors:** Idan Roifman, Nilesh R Ghugre, Mohammad I Zia, Graham A Wright, Kim A Connelly

**Affiliations:** Medicine, Imaging Research Centre for Cardiac Interventions, Schulich Heart Centre, Sunnybrook Health Sciences Centre, University of Toronto, Toronto, ON Canada; Physical Sciences Platform, Sunnybrook Research Institute, Sunnybrook Health Sciences Centre, Department of Medical Biophysics, University of Toronto, Toronto, ON Canada

## Background

The World Health Organization estimates that diabetes mellitus (DM) affects 347 million people worldwide and will be the 7^th^ leading cause of death by 2030. Right ventricular (RV) myocardial infarction and subsequent dysfunction complicating STEMI is independently associated with a higher mortality. Emerging research suggests that mechanisms for RV dysfunction may be different than those governing left ventricular dysfunction. The relationship between DM and RV dysfunction is currently unknown. The primary purpose of this study was to determine whether DM is an independent predictor for the development of right ventricular dysfunction.

## Methods

106 patients post primary percutaneous coronary intervention for STEMI were enrolled in this study between the years 2009-2013. Each patient had a cardiac MRI done on a on a 1.5T scanner within 48-72 hours of admission. Cardiac function was determined using contiguous short axis slices covering the left and right ventricle acquired with a standard SSFP sequence. RV dysfunction was defined as an MRI derived RVEF <50%. Univariate analyses were performed using the chi square, fisher's exact test, t-test, or Wilcoxon rank sum test as appropriate. Subsequently, multivariable logistic regression analysis was done in order to determine if the presence of DM was independently predictive of RV dysfunction. Predictor variables with a p value <0.25 in the univariate analysis were included in the multivariable model.

## Results

Median age of our entire patient population was 58 years (IQR 53, 67). 30% of the patients had diabetes, 44% had hypertension, 42% were active smokers and 32% had dyslipidemia. Out of 99 patients for which RV data was available, 40 had RVMIs and 59 did not (see Table 1). The presence of DM was found to be associated with a significantly higher percentage of patients with RV dysfunction (45% for patients with DM vs. 22% for patients without DM, p=0.03). There was no significant difference in age, hypertension, smoking status, dyslipidemia, serum creatinine or peak CK levels between the two groups. After adjusting for other factors, presence of DM remained an independent predictor for the development of RV dysfunction (OR 2.78, 95%CI 1.12, 6.87, p=0.03, see Table 2).

**Table 1:** Univariate analyses assessing the relationship between predictor variables and RVMI

Total N=99			
	RVMI (n=40)	No RVMI (n=59)	p-value
Age in years (median, IQR)	58 (52, 68)	58 (53, 66)	0.71
Previous MI (%)	4 (10.00)	1 (1.69)	0.15
Hypertension (%)	16 (40.00)	28 (47.46)	0.46
Smoking (%)	17 (43.59)	25 (42.37)	0.91
Hyperlipidemia (%)	14 (35.00)	18 (30.51)	0.64
Serum Creatinine in umol/l (median, IQR)	78 (66.50, 88.50)	78 (67, 95)	0.75
Peak creatine kinase in standard units/l (median, IQR)	1666.50 (815, 2395)	2096 (1103, 2964)	0.25
Diabetes (%)	18 (45.00)	13 (22.03)	0.03

**Table 2:** Multivariable logistic regression analysis assessing the relationship between predictor variables and RVMI

Predictor	Odds Ratio (95%CI)	Test statistic	p-value
Omnibus Likelihood Ratio (x2 (df))		9.46 (3)	0.02
Prior MI (yes vs. no)	4.67 (0.46, 47.03)	1.71	0.19
Peak CK in standard units/l	1.0 (0.99, 1.0)	1.39	0.24
Diabetes (yes vs. no)	2.78 (1.12, 6.87)	4.88	0.03

## Conclusions

The presence of DM is an independent predictor for the development of RV dysfunction post STEMI. In fact, its presence was associated with an approximately 3 fold greater odds of developing RV dysfunction. No other major cardiovascular risk factors were independently associated with the development of RV dysfunction in our cohort.

## Funding

Research support for this project was provided by GE healthcare and the Ontario Research Fund.

